# Effects of a Nutritional Supplement (DìRelax^TM^) on Anxiety in Dogs in a Randomized Control Trial Design

**DOI:** 10.3390/ani12040435

**Published:** 2022-02-11

**Authors:** Anna Scandurra, Vincenzo Mastellone, Maria Elena Pero, Nadia Musco, Piera Iommelli, Alfredo Di Lucrezia, Andrea Malgeri, Raffaella Tudisco, Biagio D’Aniello, Laura Cortese, Pietro Lombardi

**Affiliations:** 1 Department of Biology, University of Naples Federico II, 80126 Naples, Italy; anna.scandurra@unina.it (A.S.); a.dilcurezia@hotmail.it (A.D.L.); biagio.daniello@unina.it (B.D.); 2 Department of Veterinary Medicine and Animal Productions, University of Naples Federico II, 80137 Naples, Italy; vincenzo.mastellone@unina.it (V.M.); mp3054@cumc.columbia.edu (M.E.P.); piera.iommelli@unina.it (P.I.); tudisco@unina.it (R.T.); l.cortese@unina.it (L.C.); pilombar@unina.it (P.L.); 3 Department of Pathology, Anatomy and Cell Biology, Columbia University, New York, NY 10032, USA; 4 DVM Freelance Veterinary Practitioner, 61039 San Costanzo, Italy; a.malgeri@gmail.com

**Keywords:** dog, anxiety, nutraceuticals, impossible task, C-BARQ

## Abstract

**Simple Summary:**

The effects of a nutraceutical product, DìRelax^TM^, were tested in a cohort of anxious dogs by the C-BARQ questionnaire to assess the presence of problematic behaviors, and by the impossible task paradigm, an experimental procedure to explore dogs’ cognitive performance following an expectancy frustration. Hematological and biochemical analyses showed no adverse effects. The treatment with DìRelax^TM^ showed a positive effect on the dog’s performances, with some of the behaviors appearing improved. The results suggested that DiRelax^TM^ may have some ameliorative effect on the cognitive performances of anxious dogs.

**Abstract:**

This study aimed to investigate the efficacy of DìRelax^TM^, a nutraceutical formulated to reduce anxiety in dogs, using a randomized controlled trial (RCT) design. The C-BARQ questionnaire, some clinical investigations, and the impossible task test were performed in dogs before and after treatment. The C-BARQ questionnaire is particularly useful for assessing the frequency and severity of problematic behaviors. The impossible task paradigm provides insight into the decision-making processes in the realm of expectancy frustration. Results showed an ameliorative effect on the performances of treated dogs during the solvable phases, with a significant decrease in the time needed to solve the task. No behavioral difference was found between treated and untreated anxious dogs during the unsolvable phase. According to the results from the C-BARQ questionnaire, some of the behaviors appeared to improve. Clinical investigations, including a complete blood cell count and blood chemistry, showed no difference between groups, thus suggesting the safety of the product. In general, this study suggests that DìRelax^TM^ can be safely administered with no adverse effects and can exercise a beneficial effect on anxious dogs by enhancing their cognitive abilities, but further studies should investigate the best method of administration.

## 1. Introduction

It is well known that inappropriate behaviors could represent a serious problem that menaces the physical and psychological integrity and welfare of the dog but also of the people around it [1,2,3]. A link was proposed between stress and the beginning of anxiety [4], which is considered an important psychological disorder in dogs [5]. Anxiety is a condition induced when an environmental stimulus is improperly perceived as dangerous or threatening, and it becomes pathological when it is continued or occurs without environmental conditions justifying it [6].

Symptoms of anxiety such as excessive vocalization, destructive behavior, restlessness, inappropriate defecation and urination, hyper-salivation, and escape attempts appear when a dog is exposed to unfamiliar persons or situations. The most frequent anxiety-related disorders are separation anxiety, generalized anxiety, aggressiveness, fears, phobias, and obsessive–compulsive disorders. Flannigan and Dodman [7] observed that dogs who live in a home with a single adult human were more predisposed to have separation anxiety compared with dogs from homes with multiple owners. 

Although several strategies and behavioral therapy approaches could reduce anxiety, in many cases, a pharmacological approach is requested. Benzodiazepine, in combination with fluoxetine (a selective inhibitor of serotonin reuptake), is suggested to control signs of anxiety, including fear, aggression, and separation-related problems [8].

In addition to pharmacological intervention, natural supplements may also be used, and, indeed, their use in veterinary medicine showed a sharp increase in recent years [9]. Generally, supplements have potentially fewer side effects, and their use is not contraindicated with most drugs or disease processes (e.g., renal, hepatic, or cardiac dysfunction) in dogs. DìRelax^TM^ is a commercial nutraceutical that includes 3a700 Vitamin E (RRR-alfa-tocopherol acetate), Vitamin B6 (pyridoxine hydrochloride), and several substances that have been reported as acting analgesic and sedative properties. In particular, Escholtzia [10,11], Hops [12], Whitania [13], and Passiflora [14] share anxiolytic properties, while Krill oil, rich in polyunsaturated fatty acids (PUFAs), is involved in maintaining a healthy brain and enhancing brain functions such as reactivity, attention, and cognitive performance [15], including memory and learning [16].

Apart from the patient history as reported by the owner, there are several ways to measure anxiety in dogs. One of these is the use of the Canine Behavioral Assessment and Research Questionnaire (C-BARQ), originally designed to provide dog owners and professionals with standardized evaluations of canine temperament and behavior. The C-BARQ is particularly useful for assessing the frequency and severity of problematic behaviors, such as fear and anxiety [17]. On the other hand, some validated behavioral tests allow the measurement of stressful responses. One of these is the impossible task paradigm providing insight into the decision-making processes in the realm of expectancy frustration [18]. The experimental paradigm consists of three solvable trials in which the dog could solve an easy task to obtain a reward (e.g., feed) by manipulating an apparatus, followed by an unsolvable trial in which the reward becomes unreachable. This experimental paradigm has already been used several times in dogs for studying canine social interactions with people [19,20,21]. However, Passalacqua et al. [22] demonstrated that anxiety could impair the performance of dogs on the impossible task paradigm and induce different behavioral patterns. For example, anxious dogs passed the solvable trials with a longer latency and exhibited stress-related behaviors more times than dogs without anxiety disorders during the test [22].

The purpose of this research was to investigate the effectiveness of DìRelax^TM^ (Dynamopet, Verona, Italy) in improving anxiety in dogs. To this aim, the C-BARQ questionnaire, the impossible task paradigm, as well as hematological and biochemical analyses were used. The hypothesis was that DìRelax^TM^ might be useful in the treatment of anxiety in dogs with few or no adverse effects. 

## 2. Materials and Methods

Before confirming participation in the project, the owners were informed about the purpose and duration of the study, as well as the composition of the nutraceutical. Dogs’ owners gave their consent to house the animals in adequate facilities during the behavioral test and veterinary control, to administer their dogs the DìRelax^TM^ treatment, and to participate in the C-BARQ questionnaire before and after the treatment. DìRelax^TM^ (Dynamopet, Verona, Italy) is a mixture preparation of Krill Oil (3%), Vitamin E (RRR-alfa-tocoferil acetate) 24 mg, Vitamin B6 (pyridoxine hydrochloride) 100 mg/kg, Escholtzia (*Eschscholzia californica Cham*.) 55.2 mg/kg, Hops (*Humulus lupulus* L.) 55.2 mg/kg, Withania (*Withania somnifera* L. Dunal) 55.2 mg/kg, and Passiflora (*Passiflora incarnata* L.) 8 mg/kg. The placebo was composed of vegetable glycerol and water. The experiment, including owners’ informed consent, housing, treatment, and questionnaire, was approved by the Ethical Animal Care and Use Committee of the University of Naples Federico II, (OPBA, CSV, University of Naples Federico II, PG/2021/0123753) following local and national law, regulations, and guidelines. This research avoided distress to the animals using proper clinical management.

### 2.1. Animals

Twenty-one dogs diagnosed with anxiety disorders (i.e., generalized anxiety, separation anxiety) were involved in the study. Participating dogs sample included 10 females (5 spayed) and 11 males (2 neutered) of different breeds, with ages ranging between 1 and 15 years (mean age ± SD: 5.95 ± 4.13 years; mean weight ± SD: 13.59 ± 8.77 kg), recruited from the client-owned referral population of the Veterinary Teaching Hospital, Department of Veterinary Medicine and Animal Productions (University of Naples Federico II). To be included in the trial, dogs underwent a clinical and neurological examination and blood analysis, including complete blood count, serum biochemistry, and thyroid profiles (TSH, total T4, and free T4) to assess the health status and to exclude neurological diseases and endocrinopathies that could influence dog’s behavior. None of the animals received any drug therapy during their involvement in the study. The final sample included thirteen dogs (5.46 ± 3.84 years) in the supplemented group and eight dogs (7.13 ± 4.19 years) in the placebo group (Table 1). No difference was found between the two groups for the mean age (*p* = 0.32).

### 2.2. Experimental Design

The dogs recruited from the Veterinary Teaching Hospital, as described above, were assigned to two groups following a randomized controlled trial (RCT) design (i.e., parallel trial group), according to the type of treatment they had to undergo (i.e., supplement or placebo). It was projected to balance the number of samples in the groups. The primary endpoints of the study were the improvement of cognitive abilities and behaviors reported in the C-BARQ questionnaire. None of the owners or the experimenters was aware of which group the dogs belonged to during the study, except one experimenter that was not directly involved in the experimental test or veterinary control. The information about the type of treatment (i.e., supplement or placebo) was provided to the owners and the other experimenters only at the end of the data collection period to analyze the results obtained by the two groups of dogs. The day after the first veterinary visit, the blood sample, and the behavioral test (round 1), dogs began the treatment (i.e., supplement or placebo) for a total duration of 30 days. During the treatment, they lived with their families at home, and the pet feed was administered daily by the owners, as usual. The supplement or the placebo was orally administered daily together with the dog’s usual feed at the dose of 0.5 mg/kg, as indicated by the manufacturer. After two days from the end of the treatment, the owners and the dogs came back to the university to repeat the veterinary visit, the blood collection, and the behavioral test (round 2).

### 2.3. C-BARQ Questionnaire

The C-BARQ consists of several miscellaneous items as well as 14 different categories of behavior—stranger-directed aggression, owner-directed aggression, dog-directed aggression, stranger-directed fear, non-social fear, dog-directed fear, separation-related behavior, attachment and attention-seeking, obedience, trainability, chasing, excitability, touch sensitivity, energy level, and dog rivalry. In this research, the 42 items short C-BARQ validated by Duffy et al. [23] was adopted. The C-BARQ was verbally administered to each owner by a single experimenter, blind regarding the type of treatment the dog was subjected to. For each question, the experimenter explained in detail the scale of possible answers.

### 2.4. Blood Analysis

Blood samples were collected from each dog after 12 h fasting one day before and two days after the 30 days of the treatment from the jugular vein into tubes with and without ethylenediaminetetraacetic acid (K3EDTA). The samples were immediately transported to the laboratory at the Department of Veterinary Medicine and Animal Productions, University of Naples Federico II. On the K3EDTA samples (whole blood), a complete blood count (CBC) including hematocrit (HCT), hemoglobin (HB), red blood cells (RBC), white blood cells (WBC), and platelets (PLT) counts, was performed using a semi-automatic cell counter (Genius S, SEAC Radom Group, Calenzano, Italy). In addition, May–Grünwald–Giemsa-stained blood smears were evaluated for additional information and eventual evidence of platelet clumping. The second aliquot was centrifuged at 1200× *g* for 15 min to obtain serum samples that were frozen at −80 °C. Blood chemistry analyses on serum were performed by an automatic biochemical analyzer (Autolab, AMS Corporation, Rome, Italy) using reagents from Spinreact (Girona, Spain) to determine: total proteins (TP), urea (UREA), creatinine (CREA), glucose (GLU), alanine aminotransferase (ALT), total bilirubin (BIL), alkaline phosphatase (ALP), cholesterol (CHOL) and triglycerides (TRI). For the thyroid profile performed before recruitment, an AIA-360 Automated Immunoassay Analyzer and reagents from Tosoh (San Francisco, CA, USA) were used to assay TT4 and fT4, while TSH was assayed by the Immulite^®^ 2000 Canine (Siemens Medical Solution Diagnostics, Los Angeles, CA, USA).

### 2.5. Impossible Task Test

Dogs were subjected to the impossible task test one day before (round 1) and two days after (round 2) the treatment period (i.e., 30 days). None of the dogs involved in the study had previously performed this test. All tests were conducted at the Department of Veterinary Medicine and Animal Productions (University of Naples Federico II).

#### 2.5.1. Experimental Setting

**Figure 1:**
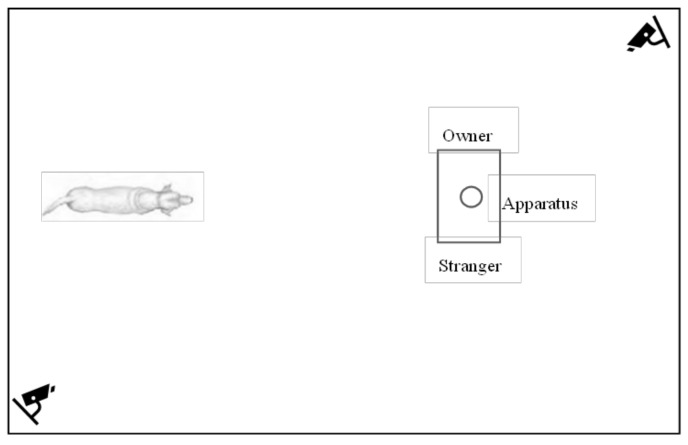
The figure shows the experimental room equipped with two cameras (in the corners) and the apparatus (in front of the dog). The owner and a stranger were placed on the two sides of the apparatus. The position of the dog in the picture indicates the starting point, about 2 meters away from the apparatus.

The tests were conducted in an empty room of about 4 × 3 m, equipped with 2 cameras, placed in two different corners of the room, and the experimental apparatus (Figure 1).

**Figure 2:**
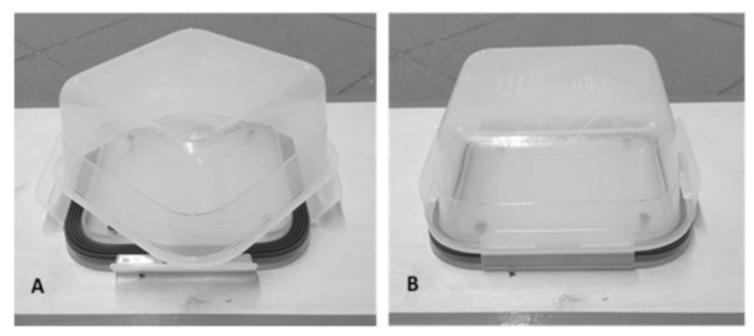
(**A**): position of feed container during the solvable phase. (**B**): locked feed container during the unsolvable phase.

The experimental apparatus consisted of a plastic feed container placed on a rectangular wooden platform. The lid of the feed container was fixed on the platform, whereas the container was placed upside down on the tracks of the lid during the solvable phase and was locked during the unsolvable phase (Figure 2).

The wooden platform was fixed on the floor by double-sided adhesive tape. All parts of the experimental apparatus were washed with a slightly perfumed and non-toxic disinfectant after each test. The feed palatability was ascertained by administering small bits of feed to the dog before the test.

#### 2.5.2. Procedure

The test consisted of three solvable trials in which the dogs could obtain the feed by manipulating the container, followed by an unsolvable trial in which the container was fixed onto the wooden board. In all the trials, the owner and an unfamiliar female person were present and maintained the identical position standing at either side of and one step back (30 cm) from the apparatus. To avoid influencing owners’ behavior during the test, the experimenter did not provide information about the specific ethogram used for the study. All participants were previously instructed by the experimenter to look straight ahead and ignored the dog (e.g., neither spoke, looked at, or touched the dog) during the test. Two different researchers were involved in the dog’s management during the test: one researcher held the dog on a starting point (e.g., denoted by an “X” on the floor; see Figure 1), and the other placed the feed below the plastic container during solvable trials, calling the dog by name to obtain the dog’s attention, and blocked the apparatus in the unsolvable trial. At the beginning of the unsolvable trial, both researchers left the room. The duration of the unsolvable trial was 60 s.

#### 2.5.3. Data Collection

The impossible task tests were video recorded and analyzed with the Solomon Coder beta^®^ 14.05.19 (ELTE TTK, Budapest, Hungary) by a blind experimenter not involved in the experimental procedure with dogs and owners. For the solvable trials, the latency of the resolution (i.e., the time in seconds from the beginning of the trial until the resolution) was observed. The behaviors of dogs during the unsolvable trial were coded according to a specific ethogram [18,20,21] (Table 2). Stress behaviors (i.e., yawning, vocalization, licking, shaking off, and scratching) were also recorded. All data were collected in frequency (number of occurrences), duration (time in seconds), and latency.

A second observer collected the same data for the inter-observer reliability. The percentage of agreement between observers results in an agreement from 90 to 100%, depending on the behavior analyzed. The data of the first observer were accepted and used for the statistical analysis.

### 2.6. Statistics

The C-BARQ data were statistically evaluated using the Wilcoxon non-parametric test.

For blood analysis, the effects of sampling times and between groups were analyzed by ANOVA according to the following model:

yijk = μ + Gi + Sj + εij

where y is the dependent variable, μ is the mean, G is the group effect (i = DìRelax^TM^, Placebo), S is the sampling effect (j = 0, 30), and ε is the error effect.

Regarding the impossible task paradigm, due to the non-normal distribution of the data, non-parametric statistics were adopted for the analysis of the C-BARQ and blood data. Comparisons were made between the two experimental groups (i.e., supplement vs. placebo) using the Mann–Whitney U-test and, in each group, between the data obtained before and after treatment (i.e., round 1 vs. round 2) using the Wilcoxon signed-rank test. All analyses were performed with GraphPad Prism^®^ software 5.01, San Diego, CA, USA.

## 3. Results

### 3.1. Clinical Scores

As depicted in Table 3, only 6 of the 42 questions from the short C-BARQ questionnaire showed significant differences between groups after the treatment.

### 3.2. Blood Analysis

Regarding hematology and blood biochemistry before and after the treatment, no statistical difference was seen between the groups, and no time effect was recorded within the groups (Table 4).

### 3.3. Impossible Task Test

**Figure 3:**
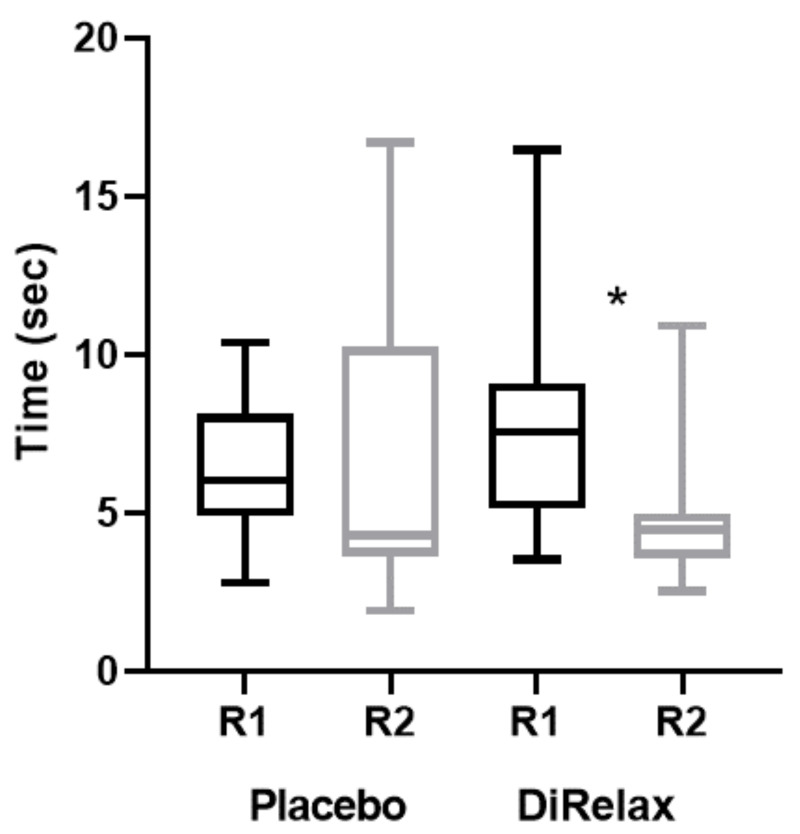
The latency of task resolution during the solvable trials in placebo and supplement (DìRelax^TM^) groups. Horizontal lines inside boxes: medians; boxes: from 25% to 75% quartiles; thin vertical lines: minimum and maximum values. Black boxes: round 1, pre-treatment; gray boxes: round 2, post-treatment. *p*-value: * *p* = 0.024.

Comparing the latencies of task resolution during the solvable phase between round 1 and round 2, it emerged that there are no significant differences in the times recorded for the dogs of the placebo group, while a significant decrease was observed in the dogs of the supplement group (Wilcoxon test, W = 72, P = 0.0024; Figure 3). No significant difference was recorded in the behaviors expressed by the dogs of both groups (supplement and placebo) during the unsolvable phase, neither towards the owner or the stranger nor in the interaction with the experimental apparatus. The two groups behaved similarly in both round 1 and round 2.

## 4. Discussion

This study evaluated the effectiveness of a commercial nutritional supplement that claims to improve anxiety in dogs. Results showed that the product might be mildly beneficial, but the administration methods should be better assessed. Dogs are the oldest domesticated animals, establishing over the years a cooperative working link with humans, sharing both foraging mode and a similar social system. Currently, they are often considered family members’ [24,25]. This intense and singular relationship reflects the way dogs and humans communicate with each other, and failing this goal negatively affects the common welfare [26,27]. Of course, anxiety negatively impacts the welfare of dogs, which in turn involves their human family. Therefore, any intervention aimed to reduce anxious responses should be welcomed. Many pharmaceutical treatments can be a helpful adjunct to behavioral modification if the animal’s fearful or anxious behavior interferes with learning or other behaviors. Most medications used to treat canine anxiety are not FDA approved for this use. Therefore, the clinician should advise the owner of any use of off-label medication and document this communication. Prior to medicating a dog, it should be examined, along with laboratory screenings conducted to evaluate its ability to metabolize and excrete the medication adequately. Use of them, such as amitriptyline or clomipramine in patients with cardiac abnormalities, seizures, or glaucoma, should be avoided if possible, or only undertaken with extreme caution, as these drugs may potentiate pre-existing cardiac conduction problems [28]. Finally, almost all of these drugs are not without side effects. For this reason, other treatments based on anxiolytic synthetic pheromones products (e.g., Adaptil^®^ dog-appeasing pheromone) have become widespread. In recent years, the use of natural remedies in alternative or together with the recommended drug therapy has been widely proposed in Western countries. Different herbal remedies are often used together to achieve several beneficial effects. Indeed, some authors suggested that a synergic effect may occur using different substances both of natural and/or industrial origin. Some nutraceuticals (e.g., Anxitane^®^, which contains the active ingredient l-theanine; or Solliquin^®^, which includes L-theanine and two plants derived extracts, *Phellodendron amurense* and *Magnolia officinalis*; and Zylkene^®^, which contains the active ingredient alpha-casozepine) and diets with some of these ingredients have a documented anti-anxiety effect, without side effects [28]. Moreover, many attempts to identify the active components of herbal remedies have concluded that, in general, no one component is responsible for the therapeutic capacity, but rather a complex and intricate interaction of various herbs may result in therapeutic efficacy [29,30]. Recently, DìRelax^TM^ has been part of the nutraceutical products and has a proven clinical efficacy (BIBL) without presenting side effects. The results of the current study seem to underline this aspect, showing an ameliorative effect during the solvable part of the impossible task paradigm.

### 4.1. Impossible Task Test

Particularly, after the treatment with the nutraceutical DìRelax^TM^, the dogs in our study showed a significant decrease in the latency of the task resolution during the solvable trials. In the previous study in the impossible task paradigm [22], anxious dogs showed a lower ability to solve the task, greater dependence on the human, as well as avoidance behaviors towards the task compared to non-anxious dogs. The significant difference found in the ability to resolve during the solvable task could indicate a relationship between cognitive performance and problems due to anxiety disorders, as demonstrated in other species [31,32]. The intake of the feed supplement may have led to improvements in the anxiety, allowing dogs to reduce the time to solve the task and to reach the reward earlier. On the other hand, no differences emerged when the feed reward was not attainable. This suggests that, although the supplement was effective in improving dogs’ cognitive performance, the improvement was not strong enough to afford the absence of reward. Together with our testing procedure, an ameliorative effect was also perceived by the owners. Indeed, the analysis of the questionnaire score showed significant favorable responses to the treatment in six questions.

### 4.2. Blood Analysis

Importantly, the lack of differences in hematology and biochemical chemistry shows that DìRelax^TM^ was well tolerated and can be safely administered to dogs using the protocol recommended by the manufacturer. Unintended effects were not found in this study.

## 5. Limitations

There is a lack of studies comparing the effectiveness of currently available nutraceuticals. The comparison between DìRelax^TM^ and other available natural anxiety treatments is therefore impossible. In our experimental design, dogs with different types (i.e., generalized, separation anxiety) and levels (i.e., moderate, severe) of anxiety disorders were involved and were randomly assigned to a specific treatment (i.e., supplement or placebo). In the future, it will be useful to evaluate the effects of the nutraceutical, specifically considering the type of anxiety disorder and the level of anxiety. The sample size of our sample could limit the external validity, although some precautions have been observed. During the study, the dogs experienced usual situations (e.g., a veterinary visit and a normal home routine), and all owners involved in the study were unaware of which treatment (i.e., supplement or placebo) they were giving their dog. This should allow a good degree of replicability of the data.

## 6. Conclusions

Taken together, the results suggest that DìRelax^TM^ possesses beneficial effects, although fairly, in improving anxiety in dogs. No adverse effects on learning or physiology were observed. However, further studies using higher doses and/or longer administration should be performed to explore a possible higher efficacy and long-lasting effects of this natural supplement.

## Figures and Tables

**Table 1:** Dogs involved in the study.

Name	Age (Years)	Breed	Weight (Kg)	Sex	Neutered	Treatment
Zikri	7	Mixed	9.1	Female	Yes	Placebo
Toki	2	Pitbull	25	Male	No	Placebo
Cliff	7	Poodle	9	Male	Yes	Placebo
Kiko	6	Shiba-inu	14.5	Male	No	Placebo
Marek	15	Beagle	15	Male	No	Placebo
Flora	11	Yorkshire	3.5	Female	No	Placebo
Molly	6	Jack Russel	5.5	Female	Yes	Placebo
Nate	3	Mixed	20	Male	No	Placebo
Baby	1	Mixed	8.6	Female	No	Supplement
Maya	3	Cocker spaniel	15	Female	No	Supplement
Laika	13	Mixed	19	Female	Yes	Supplement
Renè	2	Italian wolf	25	Male	No	Supplement
Wilma	7	Jack Russel	7.8	Female	Yes	Supplement
Rudy	2	Mixed	16	Male	Yes	Supplement
Luna	2	Mixed	8	Female	Yes	Supplement
Jack	7	Golden retriever	41	Male	No	Supplement
Joe	1	Golden retriever	17	Male	No	Supplement
Nerone	1	Mixed	7.5	Male	No	Supplement
Leo	2	Poodle	3	Male	No	Supplement
Olivia	6	Mixed	7	Female	No	Supplement
Maggie	6	Mixed	9	Female	No	Supplement

**Table 2:** Ethogram adopted for the analysis of dog’s behavior.

Target	Behavior	Description
Apparatus, Owner, Stranger	Gaze	Look at the target from a stationary position
	Interact	Physical interaction with the target
	Towards	Go toward the target

**Table 3:** Improved behaviors according to the C-BARQ questionnaire between groups. See the C-BARQ questions in the Supplemental Material for details on each section and specific behaviors.

Section	Question	*p*
Aggression	When his/her feed is taken away by a household member	0.004
Aggression	When approached while eating by another (familiar) household dog	0.050
Aggression	When barked, growled, or lunged at by an unfamiliar dog	0.023
Fear and anxiety	When groomed or bathed by a household member	0.010
Attachment and attention-seeking	Tends to follow you (or other members of the household) about the house, from room to room	0.024
Miscellaneous problems	Urinates when left alone at night or during the daytime	<0.001

**Table 4:** Complete blood count and serum biochemistry in placebo and treated dogs before and after the treatment.

	HCT	HB	RBC	WBC	PLT	TP	UREA	CREA	GLU	ALT	BIL	ALP	CHOL	TRI
	%	g/dL	10^6^/µL	10^3^/µL	10^3^/µL	g/dL	mg/dL	mg/dL	mg/dL	U/L	mg/dL	U/L	mg/dL	mg/dL
Placebo	50.16	17.19	7.185	11.11	287.67	6.42	33.62	1.32	79.93	35.12	0.200	92.94	171.62	64.06
DìRelax^TM^	47.02	16.32	6.847	10.12	255.54	6.50	35.77	1.12	74.53	38.27	0.209	72.04	185.73	55.11
Group effect p	0.083	0.220	0.078	0.412	0.079	0.767	0.267	0.140	0.153	0.408	0.760	0.056	0.239	0.111
Time effect p	0.238	0.222	0.177	0.600	0.573	0.452	0.959	0.227	0.366	0.402	0.711	0.839	0.833	0.340
RMSE	5.400	2.201	0.587	3.81	56.18	0.671	6.00	0.414	11.66	11.85	0.095	33.22	37.16	17.27

HCT: hematocrit; HB: hemoglobin; RBC: red blood cells; WBC: white blood cells; PLT: platelets; TP: total proteins; CREA: creatinine; GLU: glucose; ALT: alanine aminotransferase; BIL: total bilirubin; ALP: alkaline phosphatase; CHOL: cholesterol; TRI: triglycerides. RMSE: root mean square error.

## Data Availability

The data presented in this study are available on request from the corresponding author.
